# Longitudinal modeling of red blood cell distribution width dynamics and mortality risk in critically Ill patients with sepsis-associated acute kidney injury

**DOI:** 10.1371/journal.pone.0333605

**Published:** 2025-10-08

**Authors:** Riming He, Yijiao Liao, Ling Men, Jiahui Liu, Zhongtang Li, Ruopu Xue, Jiabing Lu, Kun Bao, Youjia Zeng, Shudong Yang

**Affiliations:** 1 Department of Nephrology, Shenzhen Traditional Chinese Medicine Hospital, The Fourth Clinical Medical College of Guangzhou University of Chinese Medicine, Shenzhen, China; 2 State Key Laboratory of Dampness Syndrome of Chinese Medicine, The Second Affiliated Hospital of Guangzhou University of Chinese Medicine, Guangzhou, China; 3 Guangdong Provincial Key Laboratory of Chinese Medicine for Prevention and Treatment of Refractory Chronic Disease, The Second Affiliated Hospital of Guangzhou University of Chinese Medicine, Guangzhou, China; 4 Department of Nephrology, Guangdong Provincial Hospital of Chinese Medicine, Guangzhou, China; University of Diyala College of Medicine, IRAQ

## Abstract

**Background:**

Sepsis-associated acute kidney injury (SA-AKI) is a critical condition with high mortality. Red cell distribution width (RDW) has emerged as a potential dynamic biomarker, but longitudinal RDW changes in SA-AKI remain underexplored.

**Methods:**

This retrospective cohort study analyzed adult SA-AKI patients from the MIMIC-IV database (2008–2022). Group-Based Trajectory Modeling (GBTM) identified distinct longitudinal RDW patterns. Primary outcome was 28-day all-cause mortality. Secondary outcomes included 90-day mortality, continuous renal replacement therapy (CRRT) requirement, and ICU length of stay. Multivariable Cox models assessed associations. adjustment for any transfusion and any major hemorrhage, and an exclusion analysis in which all transfused patients were removed.

**Results:**

Among 6,694 patients (mean age 65.5 years, 57.7% male), 28-day mortality was 22.5%. Four RDW trajectory groups were identified: Stable Low (27.8%), Gradual Increase (38.5%), Continuous Increase (27.6%), and Rapid Increase (6.1%). The Rapid Increase group demonstrated highest disease severity scores and poorest laboratory profiles. Compared to the Stable Low group, the Rapid Increase group had significantly elevated 28-day mortality risk after full adjustment (HR 4.27, *P* < 0.001), with consistent patterns for 90-day mortality and resource use. Associations remained consistent across subgroup analyses, multiple-imputation datasets, and the relaxed-inclusion cohort, and were minimally altered after adjusting for transfusion/hemorrhage or excluding transfused patients.

**Conclusions:**

Dynamic RDW trajectories were independently associated with adverse outcomes in SA-AKI patients. Across extensive sensitivity analyses, these trajectories functioned as dynamic prognostic indicators without implying causality, supporting their use for risk stratification and individualized patient care.

## Introduction

Sepsis, characterized by dysregulated host responses to infection, frequently results in acute kidney injury (AKI), specifically termed sepsis-associated acute kidney injury (SA-AKI) [1]. SA-AKI is linked to an increased risk of chronic kidney disease (CKD), prolonged hospitalization, and elevated mortality rates compared to AKI without sepsis [2,3]. The pathophysiology of SA-AKI is multifaceted, encompassing inflammation, endothelial dysfunction, and microcirculatory disturbances, which contribute to the observed high mortality rates [4].

In the pursuit of enhanced prognostic markers for critically ill patients, red blood cell distribution width (RDW) has been identified as a potential biomarker. RDW, a metric of red blood cell size variability, traditionally differentiates anisocytosis in various anemias [5]. Recent research suggests RDW’s involvement in the pathogenesis of multiple organ dysfunction syndromes, including AKI [6–9]. RDW correlates with inflammation, oxidative stress, and malnutrition-crucial factors in sepsis progression and its complications [10]. RDW also emerges as an independent mortality predictor in critically ill patients, including those with sepsis [11]. Despite the established significance of RDW as a prognostic marker in SA-AKI [11,12], systematic investigations into its longitudinal patterns and correlation with mortality risk are scarce. Prior research, often limited to single or short-term assessments [12], has not comprehensively delineated the long-term trajectory of RDW, nor has it sufficiently clarified the prognostic implications of varying RDW escalation patterns.

In this study, we extracted SA-AKI patients from the Medical Information Mart for Intensive Care (MIMIC)-Ⅳ public database who fulfilled specific diagnostic and exclusion criteria. Utilizing Group-Based Trajectory Modeling (GBTM), a person-centered analytical approach [13,14], we conducted a segmented analysis of the longitudinal changes in RDW during the ICU stay. Firstly, by ascertaining the optimal number and form of trajectory subgroups, we investigated the dynamic patterns of RDW evolution throughout the disease course. Secondly, employing a multivariable Cox regression model, we evaluated the correlations between distinct RDW trajectory groups and outcomes, including 28-day and 90-day all-cause mortality, the use of continuous renal replacement therapy (CRRT), and ICU length of stay, with the goal of offering novel evidence for early clinical risk stratification and personalized therapeutic strategies.

## Methods

### Data sources

This retrospective cohort study was conducted using data from the Medical Information Mart for Intensive Care IV (MIMIC-IV) database, version 3.0, which contains comprehensive electronic health records from patients admitted to the intensive care units of Beth Israel Deaconess Medical Center, Boston, Massachusetts, from 2008 to 2022 [15]. All patient data in the database have been de-identified in accordance with the Health Insurance Portability and Accountability Act (HIPAA) privacy rule, eliminating the need for individual patient consent [16]. Access to the database was granted following completion of the Collaborative Institutional Training Initiative (CITI) program conducted by the National Institutes of Health (Record ID: 38913928). Data extraction and initial SQL queries for this study were performed on 01 December 2024. All datasets were fully de-identified before release; the investigators had no access to any direct or indirect patient identifiers at any stage of the analysis.

The MIMIC-IV project has existing approvals from the Institutional Review Boards of Beth Israel Deaconess Medical Center and the Massachusetts Institute of Technology, both granting a waiver of informed consent. Because only publicly available, de-identified data were used, the present study required no additional IRB review.

We report all ethics/provenance information solely in the Methods as per journal policy. All SQL extraction queries, data cleaning scripts, and analysis code are publicly available at GitHub: https://github.com/heriming13/RDW_Trajectory_SAAKI.

### Population selection criteria

Adult patients (≥18 years) with sepsis-associated acute kidney injury (SA-AKI) were included in the analysis. SA-AKI was defined according to the consensus definition established by the 28th Acute Disease Quality Initiative (ADQI) working group, which requires the occurrence of AKI within a 7-day window following sepsis onset, consistent with both Sepsis-3 and Kidney Disease: Improving Global Outcomes (KDIGO) criteria [17]. Sepsis was identified using the Sepsis-3 criteria, requiring the presence or suspicion of infection accompanied by a Sequential Organ Failure Assessment (SOFA) score ≥2 points [18]. Infections were confirmed through International Classification of Diseases, Ninth and Tenth Revision (ICD-9 and ICD-10) diagnostic codes recorded in the MIMIC-IV database. Suspected infections were identified by the administration of empirical antibiotic therapy within a 3-day window (before or after) of microbiological culture sample collection, as previously validated in critical care databases. AKI was diagnosed based on the KDIGO criteria, requiring one or more of the following conditions: an increase in serum creatinine (SCr) of ≥26.5 μmol/L (0.3 mg/dL) within 48 hours, an increase in SCr to ≥1.5 times the baseline value within 7 days, or urine output <0.5 mL/kg/hour for ≥6 consecutive hours [19]. Baseline serum creatinine was defined as the lowest SCr value measured within 7 days prior to hospital admission, or the admission SCr value if no pre-admission measurements were available [20].

Patients were excluded if they met any of the following criteria: AKI developed prior to sepsis diagnosis or >7 days after sepsis onset, continuous renal replacement therapy (CRRT) initiated before AKI diagnosis, history of AKI before current ICU admission, death within 96 hours of ICU admission, ICU length of stay <96 hours, fewer than 5 RDW measurements during the 28-day follow-up period, or multiple ICU admissions during the study period (only the first admission was analyzed).

For sensitivity analyses, we additionally constructed a relaxed cohort requiring only ≥3 RDW measurements and removing the ICU length-of-stay ≥96 h restriction; trajectory modeling and outcome analyses were repeated identically in this cohort (see Statistical Analysis).

### Data collection

Data extraction from the MIMIC-IV database was performed using Structured Query Language (SQL) through the pgAdmin4 interface for PostgreSQL database management. Demographic and clinical variables collected included age, sex, race, body mass index (BMI), and mean arterial pressure (MAP) recorded within 24 hours of ICU admission, along with comorbidities including hypertension, diabetes mellitus, heart failure, malignant tumors, chronic obstructive pulmonary disease (COPD), and liver cirrhosis. Disease severity was assessed using the Sequential Organ Failure Assessment (SOFA) score, Acute Physiology Score III (APS III), Oxford Acute Severity of Illness Score (OASIS), and Charlson Comorbidity Index (CCI). All severity scores were calculated using the worst values within the first 24 hours after ICU admission, preceding the longitudinal RDW trajectory window used for exposure assignment. Laboratory values obtained within 24 hours of ICU admission included complete blood count parameters (white blood cell count, hemoglobin, hematocrit, red blood cell distribution width, platelet count), serum chemistry markers (albumin, sodium, potassium, total calcium, chloride, bicarbonate, total bilirubin, creatinine), and arterial blood gas measurements (pH, lactate). RDW values were collected longitudinally throughout the 28-day follow-up period, with only the first measurement of each calendar day included to minimize measurement bias and ensure temporal consistency. A minimum of 5 RDW measurements during the 28-day period was required for trajectory analysis. Details of missing data handling are described under Statistical Analysis, with additional information provided in the Supplementary Materials.

Transfusion and hemorrhage variables (for confounding control) were identified using database-specific codes and item labels in MIMIC-IV: we created binary indicators for any blood transfusion (packed red blood cells, plasma, platelets) and any major hemorrhage during the ICU stay. These indicators were incorporated into adjusted models and used to define an exclusion subset (patients with any transfusion removed) in sensitivity analyses.

### Outcomes

The primary outcome was 28-day all-cause mortality following ICU admission. Secondary outcomes included 90-day all-cause mortality, requirement for continuous renal replacement therapy (CRRT), and length of ICU stay.

### Statistical analysis

We employed Group-Based Trajectory Modeling (GBTM), a person-centered analytical approach, to identify distinct longitudinal patterns of RDW changes during the ICU stay [13,14]. This semi-parametric mixture modeling technique allows for the identification of homogeneous subgroups within a heterogeneous population based on similar developmental trajectories over time. The optimal number of trajectory groups was determined through systematic evaluation of models with 2–5 latent classes, with model selection based on multiple statistical criteria including Akaike Information Criterion (AIC), Bayesian Information Criterion (BIC), Sample size-adjusted BIC (SABIC), entropy values, log-likelihood ratios, and clinical interpretability. For each potential trajectory shape (linear, quadratic, and cubic), we evaluated model fit statistics and selected the optimal configuration. The final model was required to meet the following criteria: each identified trajectory group comprised >5% of the study population, average posterior probability of group membership >0.7 for each trajectory, and adequate model convergence and clinical meaningfulness [21]. The analysis adhered to the Guidelines for Reporting on Latent Trajectory Studies (GRoLTS) checklist to ensure methodological rigor and transparency [22], our analysis identified four distinct trajectories as the optimal model fit (Tables S2 and S3 in S1 File).

Sensitivity analyses for selection bias and robustness included: (1) a relaxed inclusion cohort requiring ≥3 RDW measurements and removing the ICU LOS ≥ 96 h restriction, in which trajectory modeling and Cox analyses were replicated; (2) models additionally adjusting for any transfusion and any major hemorrhage, and models excluding all transfused patients; and (3) models excluding severity scores (SOFA, APS III, OASIS) from the adjustment set to assess potential over-adjustment/collider bias.

Continuous variables were assessed for normality using multiple methods including histogram visualization, quantile-quantile plots, and the Kolmogorov-Smirnov test. Normally distributed variables were expressed as mean ± standard deviation (SD), while non-normally distributed variables were reported as median with interquartile range (IQR). Categorical variables were presented as frequencies and percentages. Between-group differences were assessed using chi-square test for categorical variables, one-way analysis of variance (ANOVA) for normally distributed continuous variables, and Kruskal-Wallis H test for non-parametric continuous variables. For multiple comparisons, p-values were adjusted using Bonferroni correction, Tukey’s honestly significant difference, or least significant difference (LSD) methods to control the type I error rate. Kaplan-Meier survival curves were constructed to evaluate all-cause mortality differences among RDW trajectory groups, with statistical significance assessed using the log-rank test.

The association between RDW trajectory groups and 28-day all-cause mortality was evaluated using Cox proportional hazards regression models. Three progressively adjusted models were constructed: Model 1 (unadjusted model with RDW trajectory as the sole predictor), Model 2 (adjusted for demographic variables including age, sex, and race), and Model 3 (fully adjusted model including all clinically relevant confounders). Confounding variables for Model 3 were selected based on clinical relevance and biological plausibility, previous literature evidence, statistical significance in univariate analysis, variables associated with both exposure and outcome, and covariates that modified effect estimates by >10%. The final adjustment set included BMI, MAP, SOFA score (worst within 24 h), APS III (worst within 24 h), OASIS (worst within 24 h), CCI, comorbidities (hypertension, diabetes mellitus, heart failure, malignant tumors, COPD, cirrhosis), and laboratory parameters (WBC, hemoglobin, hematocrit, platelet count, albumin, electrolytes, pH, lactate, total bilirubin, creatinine, bicarbonate). Trend analysis was performed using linear regression.

To quantify selection differences, we compared baseline characteristics between the included cohort (≥5 RDW; ICU LOS ≥ 96 h by design) and excluded/early-death patients (<5 RDW or ICU LOS ≤ 96 h) using standardized mean differences (ASD); an ASD < 0.10 was interpreted as negligible imbalance.

Pre-specified subgroup analyses were conducted to assess the consistency of associations across different patient populations, with subgroups defined based on age (<65 vs ≥ 65 years), sex (male vs female), BMI tertiles, and major comorbidities (hypertension, diabetes mellitus, heart failure, COPD). Interaction effects between trajectory groups and subgroup variables were tested using likelihood ratio tests. To address potential bias from missing data, multiple imputation was performed using predictive mean matching for continuous variables, logistic regression for binary variables, and multinomial logistic regression for categorical variables. Five imputed datasets were generated using sequential chained equations, and results were pooled using Rubin’s rules [23]. The consistency between complete case analysis and multiple imputation results was evaluated to assess the robustness of findings.

All statistical analyses were performed using R Statistical Software (version 4.2.2) and the Free Statistics Analysis Platform (version 2.0). A two-tailed p-value <0.05 was considered statistically significant. Hazard ratios (HR) and regression coefficients were reported with 95% confidence intervals (CI). This study was conducted in accordance with the Declaration of Helsinki and approved by the institutional review board. The use of the MIMIC-IV database for research purposes is exempt from additional ethical review due to its publicly available and de-identified nature, and no additional patient consent was required for this retrospective analysis.

## Results

### RDW trajectory groups

**Fig 1:**
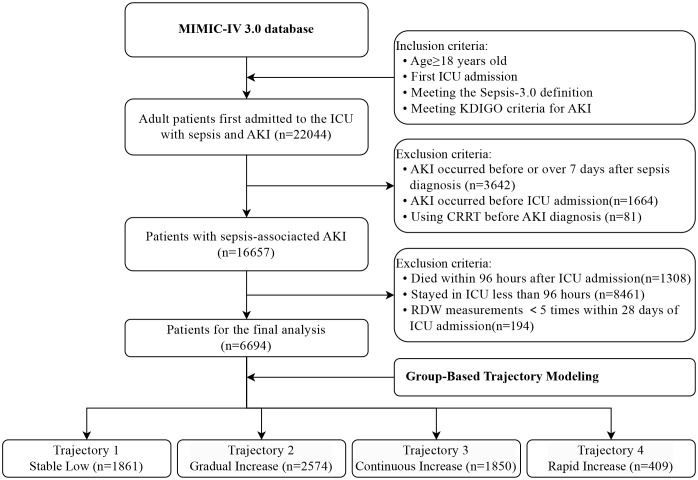
Flow charts of the study populations.

**Fig 2:**
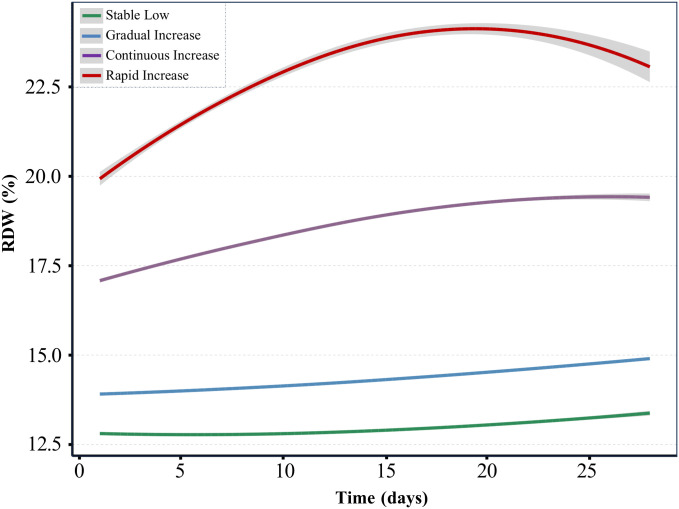
Trajectories of RDW during follow-up visits. We identified four distinct trajectories of Red Cell Distribution Width (RDW) changes during the 28-day follow-up period through group-based trajectory modeling (GBTM). The solid lines represent the average predicted levels of specific categories, serving as a function of follow-up estimated by the best-fitting model, and the shaded areas around the solid lines indicate the confidence intervals for the calculated trajectories.

A total of 6,694 SA-AKI patients met the inclusion criteria (Fig 1). Utilizing a GBTM, we identified four trajectory classes of RDW among AKI patients during their ICU stay (Fig 2), with model fit details provided in Tables S2 and S3 in S1 File. The trajectories were labeled based on initial levels and trends as follows: 1) Stable Low trajectory group (n = 1,861, 27.8%), characterized by consistently low RDW levels; 2) Gradual Increase trajectory group (n = 2,574, 38.5%), exhibiting a slow yet persistent rise in RDW; 3) Continuous Increase trajectory group (n = 1,850, 27.6%), with a continuous upward trend in RDW; and 4) Rapid Increase trajectory group (n = 409, 6.1%), featuring a sharp initial increase in RDW followed by stabilization. The posterior probabilities for each group were 79%, 71%, 79%, and 86%, respectively (Table S3 in S1 File).

In a pre-specified sensitivity cohort with relaxed inclusion (≥3 RDW measurements and without the ICU length-of-stay ≥96 h restriction), the 4-class structure was reproduced with comparable shapes and class proportions, supporting the robustness and parsimony of the chosen solution (Table S4 in S1 File; Fig S1 in S1 File). Although a 5-class model provided a slightly lower BIC in that cohort, the minimum class proportion was small and entropy gains were limited; thus, we retained 4 classes for clinical interpretability.

### Baseline characteristics according to RDW trajectories

As shown in Table 1, among the 6,694 eligible participants, the average age at baseline was 65.5 years, with a male predominance of 57.7%. Compared to other groups, the Rapid Increase group was younger, had the lowest proportion of white participants, and presented with the highest disease severity scores (SOFA, APS III, OASIS, and CCI). Laboratory parameters revealed significant differences in hemoglobin, hematocrit, baseline RDW, PLT, albumin, and electrolyte levels across groups. Notably, the Rapid Increase group had lower albumin levels (2.8 g/dL), indicating poorer nutritional status (**P* *< 0.001), and higher levels of lactate, total bilirubin, and creatinine, suggesting more severe metabolic acidosis and impaired liver and kidney function (all *P* < 0.001). The primary outcome of interest, 28-day all-cause mortality, increased significantly from the Stable Low group (12.7%) to the Rapid Increase group (53.3%) (*P* < 0.001). This trend was consistent with 90-day mortality, rising from 17% in the Stable Low group to 60.9% in the Rapid Increase group (*P* < 0.001). The need for CRRT and ICU length of stay also increased with worsening RDW trajectories, highlighting the severity of these patients’ conditions (both *P* < 0.001).

**Table 1:** Baseline characteristics according to RDW trajectories.

Variables	Total	RDW trajectory groups				*P*-value
		Stable Low (n = 1861)	Gradual Increase (n = 2574)	Continuous Increase (n = 1850)	Rapid Increase (n = 409)	
**Demographic data**						
Age (years)	65.5 ± 16.3	63.2 ± 17.6	67.2 ± 15.7	66.0 ± 15.6	62.6 ± 15.6	< 0.001
Male (%)	3865 (57.7)	1226 (65.9)	1506 (58.5)	933 (50.4)	200 (48.9)	< 0.001
Race_white (%)	4159 (62.1)	1133 (60.9)	1638 (63.6)	1142 (61.7)	246 (60.1)	0.21
BMI (kg/m^2^)	30.1 ± 8.5	29.4 ± 7.2	30.5 ± 8.5	30.2 ± 9.4	30.0 ± 8.5	0.002
MAP (mmHg)	83.7 (72.3, 97.0)	87.7 (76.0, 100.3)	83.0 (71.7, 96.7)	80.7 (70.3, 93.2)	81.7(70.3, 93.8)	< 0.001
**Severity scoring**						
SOFA	6.8 ± 3.7	5.4 ± 3.1	6.7 ± 3.4	7.8 ± 3.8	9.3 ± 4.1	< 0.001
APS Ⅲ	55.0 ± 22.5	46.2 ± 19.4	54.4 ± 21.6	61.5 ± 22.8	69.0 ± 23.7	< 0.001
OASIS	36.8 ± 8.2	34.9 ± 7.7	37.1 ± 7.9	37.9 ± 8.6	37.9 ± 8.3	< 0.001
CCI	5.2 ± 2.9	4.2 ± 2.8	5.2 ± 2.8	5.9 ± 2.9	6.0 ± 3.0	< 0.001
**Comorbidities (%)**						
Hypertension	2790 (41.7)	926 (49.8)	1089 (42.3)	656 (35.5)	119 (29.1)	< 0.001
Diabetes mellitus	2041 (30.5)	492 (26.4)	832 (32.3)	608 (32.9)	109 (26.7)	< 0.001
Heart failure	2116 (31.6)	411 (22.1)	865 (33.6)	697 (37.7)	143 (35)	< 0.001
Malignant tumors	919 (13.7)	218 (11.7)	359 (13.9)	284 (15.4)	58 (14.2)	0.014
COPD	656 (9.8)	147 (7.9)	271 (10.5)	200 (10.8)	38 (9.3)	0.01
Cirrhosis	641 (9.6)	21 (1.1)	135 (5.2)	319 (17.2)	166 (40.6)	< 0.001
**Laboratory tests**						
WBC (k/μL)	12.1 (8.6, 16.9)	12.2 (9.0, 16.1)	12.1 (8.5, 16.6)	12.1 (8.1, 17.7)	12.8(8.1, 18.8)	0.445
Hemoglobin (g/dL)	10.8 ± 2.3	12.1 ± 2.1	10.8 ± 2.2	9.9 ± 2.1	9.2 ± 2.1	< 0.001
Hematocrit (%)	32.9 ± 7.0	36.2 ± 6.3	32.8 ± 6.8	30.8 ± 6.6	28.6 ± 6.4	< 0.001
RDW (%)	15.2 ± 2.3	13.3 ± 0.7	14.7 ± 1.0	16.7 ± 2.0	19.5 ± 3.7	< 0.001
PLT (k/μL)	203.3 ± 111.6	211.0 ± 89.4	206.2 ± 106.8	197.2 ± 127.3	177.2 ± 146.4	< 0.001
Albumin (g/dL)	2.9 ± 0.6	3.2 ± 0.6	2.9 ± 0.6	2.7 ± 0.6	2.8 ± 0.6	< 0.001
Sodium (mmol/L)	138.6 ± 5.8	138.8 ± 5.1	139.0 ± 5.7	138.4 ± 6.0	137.0 ± 7.3	< 0.001
Potassium (mmol/L)	4.2 ± 0.8	4.1 ± 0.7	4.2 ± 0.8	4.3 ± 0.8	4.2 ± 0.9	< 0.001
tCa (mg/dL)	8.2 ± 0.9	8.3 ± 0.8	8.2 ± 0.9	8.1 ± 1.0	8.0 ± 1.0	< 0.001
Chloride (mmol/L)	104.3 ± 7.1	104.4 ± 6.1	104.9 ± 7.0	103.8 ± 7.5	102.2 ± 8.8	< 0.001
pH	7.3 ± 0.1	7.4 ± 0.1	7.3 ± 0.1	7.3 ± 0.1	7.3 ± 0.1	< 0.001
Lactate (mmol/L)	1.8 (1.2, 2.9)	1.7 (1.2, 2.5)	1.8 (1.2, 2.9)	1.9 (1.3, 3.1)	2.5 (1.6, 4.2)	< 0.001
Tbil (mg/dL)	0.7 (0.4, 1.4)	0.6 (0.4, 0.9)	0.6 (0.4, 1.0)	0.8 (0.4, 2.1)	2.0 (0.8, 7.3)	< 0.001
Creatinine (mg/dL)	1.0 (0.8, 1.6)	0.9 (0.7, 1.2)	1.1 (0.8, 1.6)	1.2 (0.8, 2.0)	1.3 (0.9, 2.1)	< 0.001
Bicarbonate (mmol/L)	22.3 ± 5.1	23.0 ± 4.3	22.4 ± 4.9	21.9 ± 5.6	20.3 ± 5.5	< 0.001
**CRRT (%)**	759 (11.3)	54 (2.9)	243 (9.4)	354 (19.1)	108 (26.4)	< 0.001
**Length of ICU stay (days)**	7.9 (5.5, 12.8)	7.4 (5.3, 11.6)	7.8 (5.4, 13.1)	8.2 (5.7, 13.3)	8.6 (5.8, 13.1)	< 0.001
**28-day mortality**	1505 (22.5)	237 (12.7)	489 (19)	561 (30.3)	218 (53.3)	< 0.001
**90-day mortality**	1982 (29.6)	316 (17)	660 (25.6)	757 (40.9)	249 (60.9)	< 0.001

Note: Continuous variables that were normally distributed were reported as mean ± standard deviation (SD), while skewed continuous variables were described as median with interquartile range (IQR). Categorical variables were presented as frequencies and percentages (%).

Abbreviations: MAP, mean arterial pressure; SPO2, saturation of peripheral oxygen; SOFA, sequential organ failure assessment score; APS Ⅲ, acute physiology score Ⅲ; OASIS, Oxford acute severity of illness score; CCI, Charlson comorbidity index; COPD, chronic obstructive pulmonary disease; WBC, white blood cell count; PLT, platelet count; tCa, total Calcium; Tbil, total bilirubin; CRRT, continuous renal replacement therapy.

To quantify potential selection differences arising from the main inclusion criteria (≥5 RDW; ICU LOS ≥ 96 h), we compared baseline characteristics between included patients and excluded/early-death patients (<5 RDW or ICU LOS ≤ 96 h). Several variables showed standardized differences >0.10, indicating non-negligible imbalance (Table S5 in S1 File).

### Associations between RDW trajectories and outcomes

**Fig 3:**
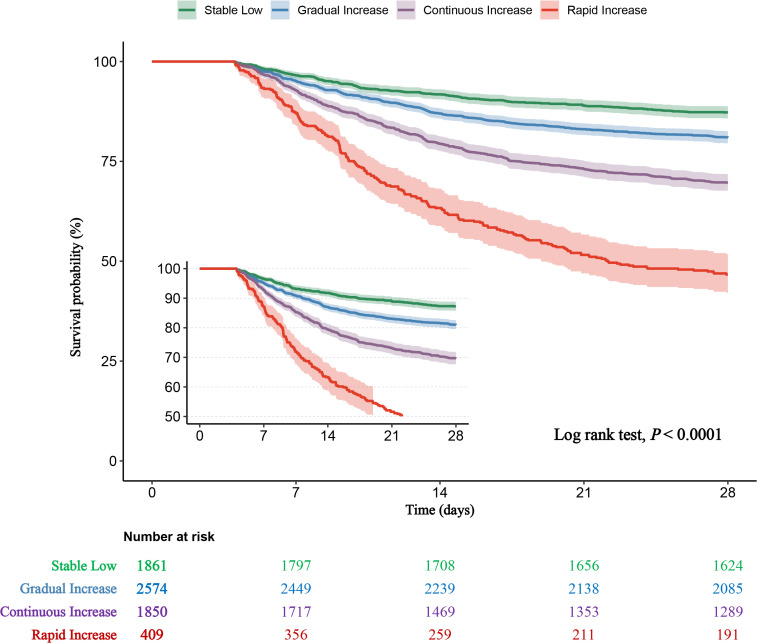
Kaplan‒Meier survival analysis curves for all-cause mortality based on trajectories of the RDW.

Kaplan-Meier survival analysis demonstrated a significant association between RDW trajectory groups and 28-day mortality risk, with the highest mortality in the Rapid Increase group (log-rank test, *P* < 0.001) (Fig 3). Cox proportional hazards modeling revealed that, compared to the Stable Low group, the Gradual Increase, Continuous Increase, and Rapid Increase groups had increased mortality risks of 55%, 164%, and 447%, respectively (HR 1.55, 2.64, 5.47, all *P* < 0.001) in the unadjusted Model 1. After adjusting for age, gender, race, and clinical and laboratory indicators in Model 3, these risks remained significant, albeit reduced (HR 1.45, 2.18, 4.27, all *P* < 0.001) (Table 2). Similar trends were observed for 90-day mortality and the need for CRRT, with the Rapid Increase group at the highest risk. ICU length of stay was also associated with RDW trajectory, being longest in the Rapid Increase group (Table 3). These findings underscore the prognostic significance of RDW as a dynamic marker of disease severity and its potential role in risk stratification and clinical decision-making.

**Table 2:** Cox proportional hazards analyses for 28-day all-cause mortality according to RDW trajectories.

Categories	Model 1		Model 2		Model 3	
	HR (95%CI)	*P*-value	HR (95%CI)	*P*-value	HR (95%CI)	*P*-value
Stable Low group	1(Ref)		1(Ref)		1(Ref)	
Gradual Increase group	1.55 (1.32-1.81)	<0.001	1.45 (1.24-1.69)	<0.001	1.45 (1.14-1.83)	0.002
Continuous Increase group	2.64 (2.27-3.07)	<0.001	2.58 (2.21-3)	<0.001	2.18 (1.71-2.78)	<0.001
Rapid Increase group	5.47 (4.55-6.57)	<0.001	5.8 (4.82-6.98)	<0.001	4.27 (3.18-5.75)	<0.001

Model 1: crude relative risk;

Model 2: adjusted for age, gender, and race (white participants or others);

Model 3: further adjusted (from Model 2) for BMI, MAP, SOFA, APS Ⅲ, OASIS, CCI, hypertension, diabetes mellitus, heart failure, malignant tumors, COPD, cirrhosis, WBC, hemoglobin, hematocrit, PLT, albumin, sodium, potassium, total calcium, chloride, pH, lactate, total bilirubin, creatinine, and bicarbonate.

**Table 3:** Multivariable regression analysis (Cox or linear) for secondary outcomes according to RDW trajectories.

Categories	90-day all-cause mortality		CRRT		Length of ICU stay	
	Adjusted HR (95%CI)^a^	*P*-value	Adjusted HR (95%CI)^a^	*P*-value	Adjusted β (95%CI)^b^	*P*-value
Stable Low group	1(Ref)		1(Ref)		0(Ref)	
Gradual Increase group	1.43 (1.16-1.75)	0.001	2.13 (1.51-3.01)	<0.001	1.46 (0.58-2.34)	0.001
Continuous Increase group	2.08 (1.69-2.56)	<0.001	3.56 (2.5-5.06)	<0.001	2.43 (1.43-3.43)	<0.001
Rapid Increase group	3.73 (2.87-4.85)	<0.001	5.55 (3.65-8.44)	<0.001	2.47 (0.94-3.99)	0.002

^a^ Adjusted HRs were calculated via the Cox proportional hazards model.

^b^ Adjusted β were calculated via the Multivariable linear regression model.

Both multivariable regression models adjusted for age, gender, race, BMI, MAP, SOFA, APS Ⅲ, OASIS, CCI, hypertension, diabetes mellitus, heart failure, malignant tumors, COPD, cirrhosis, WBC, hemoglobin, hematocrit, PLT, albumin, sodium, potassium, total calcium, chloride, pH, lactate, total bilirubin, creatinine, and bicarbonate.

### Subgroup and sensitivity analyses

**Fig 4:**
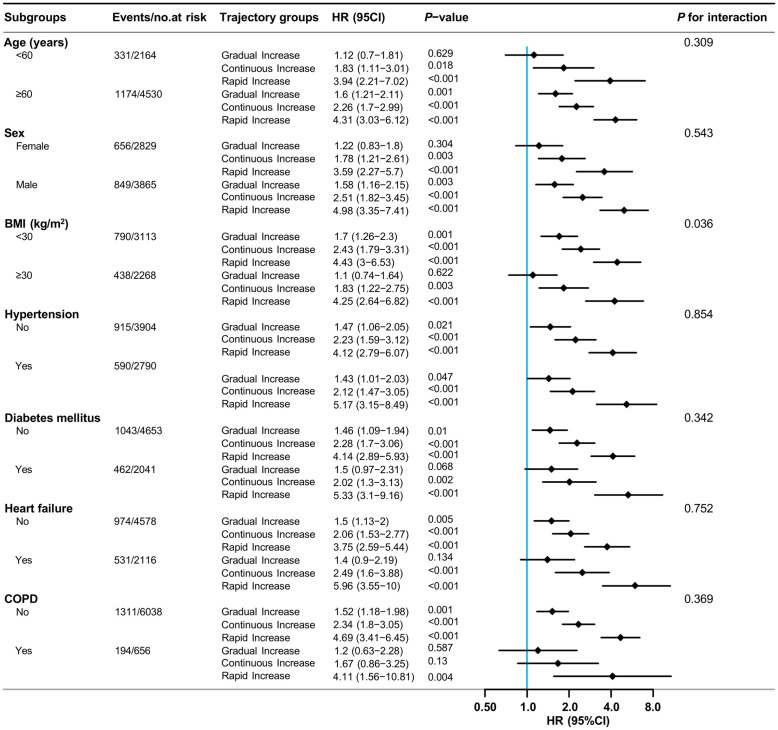
Adjusted HR (95% CIs) for 28-day all-cause mortality according to RDW trajectories in subgroups analyses*. *Results were adjusted for age, gender, race, BMI, MAP, SOFA, APS Ⅲ, OASIS, CCI, hypertension, diabetes mellitus, heart failure, malignant tumors, COPD, cirrhosis, WBC, hemoglobin, hematocrit, PLT, albumin, sodium, potassium, total calcium, chloride, pH, lactate, total bilirubin, creatinine, and bicarbonate, and stratified variables were not included in the relevant models.

Subgroup analyses, stratified by age, gender, BMI, hypertension, diabetes, heart failure, and COPD status, showed consistent associations between RDW trajectory groups and 28-day all-cause mortality risk (Fig 4). The Rapid Increase group exhibited the highest HR across all subgroups, ranging from 3.59 to 5.96, with statistical significance (*P* < 0.05). The lack of significant interaction effects across subgroups suggests a uniform impact of RDW trajectory on mortality risk.

In sensitivity analyses, comparisons of data distributions before and after multiple imputation revealed no significant differences in key clinical factors (Table S6 in S1 File). The application of Cox regression to the imputed dataset yielded results highly consistent with the initial analysis (Table S7 in S1 File), reinforcing the robustness of RDW trajectory as a significant predictor of 28-day all-cause mortality, irrespective of missing data treatment. In the relaxed criteria cohort (≥3 RDW), adjusted associations with 28- and 90-day mortality remained robust (Table S8 in S1 File). Additional sensitivity analyses addressing hematologic confounding showed that adjusting for any ICU blood transfusion and any major hemorrhage, and separately excluding all transfused patients, had minimal impact on effect estimates across trajectory groups (Table S9 in S1 File). To assess potential over-adjustment due to severity scores, models excluding SOFA/APS III/OASIS produced estimates similar in direction and magnitude to the fully adjusted models (Table S10 in S1 File).

## Discussion

In the longitudinal cohort study involving over 6,000 patients with SA-AKI, we identified four distinct trajectories of RDW over time using GBTM. Analysis with multivariable Cox regression and multiple imputation confirmed a significant association between increasing RDW and adverse outcomes. Specifically, transitions from a “stable low” to a “rapidly increasing” RDW trajectory were associated with increased 28-day and 90-day all-cause mortality, CRRT utilization, and ICU length of stay, suggesting that RDW dynamics can sensitively reflect disease progression and are associated with poor prognosis. Findings were consistent across multiple subgroup analyses and a series of prespecified sensitivity analyses (relaxed inclusion criteria; adjustment for or exclusion of transfusion or hemorrhage), supporting the robustness and generalizability of the associations. To our knowledge, this study is the first to delineate RDW trajectories in SA-AKI patients, offering a novel approach for early risk stratification and personalized intervention strategies.

Previous studies have widely reported that elevated RDW is highly associated with adverse outcomes in various acute and chronic diseases [24–26], particularly in critically ill patients, including those with sepsis [27], heart failure [28], and acute pancreatitis [29]. RDW has demonstrated prognostic association mortality risk. For instance, a recent systematic review integrating 24 clinical studies identified RDW as an independent predictor of mortality in sepsis patients [30]. Lai et al.reported a significant association between RDW and 28-day mortality in SA-AKI patients and attempted to integrate RDW values into a Nomogram for clinical personalized risk prediction [12]. However, most of these studies were limited to baseline values, and single-point RDW studies have significant limitations: they cannot reveal the dynamic evolution of RDW over time, potentially underestimating the complexity of individual disease changes, and are susceptible to acute environmental interferences such as fluid resuscitation or blood transfusion. In recent time-series analyses, including a study by Kim et al., RDW was assessed in sepsis patients at admission and again after 72 hours. The findings indicated that an acute increase in RDW correlates with increased mortality risk [11]. However, these studies only performed linear analysis on “baseline and increase amplitude,” failing to identify the heterogeneity of patient RDW change curves. Our study, with its large sample size and multiple time points, further supports the value of RDW as a prognostic marker for SA-AKI and explicitly aligns covariate timing by computing severity scores (SOFA, APS III, OASIS) within the first 24 hours after ICU admission (prior to trajectory assignment), and by confirming robustness in models excluding these scores.

The dynamic increase of RDW in SA-AKI may reflect the combined effects of multiple pathophysiological mechanisms. Inflammation and immune dysregulation are considered the primary drivers. Firstly, the “inflammatory storm” in sepsis can induce endothelial cell dysfunction and exacerbate microcirculatory disturbances, leading to any imbalance in the generation, maturation, and destruction of red blood cells, potentially widening the RBC volume distribution [31,32]. Secondly, oxidative stress and cell membrane damage also play a significant role in RDW elevation. Red blood cells are particularly sensitive to reactive oxygen species (ROS), and when excessive ROS accumulate and attack red blood cell membranes, they can cause lipid peroxidation, impair repair capacity, and accelerate the generation and release of abnormal red blood cells or immature red blood cells [33–35]. Thirdly, nutritional and metabolic imbalances are also potential causes of rapid RDW increase. On one hand, malnutrition, hypoalbuminemia, or deficiencies in trace elements (such as iron, folic acid) can hinder normal red blood cell production, leading to increased red blood cell morphological heterogeneity [31,36]. On the other hand, severe infections often accompany multi-organ failure (liver, kidney dysfunction), affecting iron homeostasis, erythropoietin (EPO) secretion, and the clearance of aged red blood cells, further widening RDW [37,38]. Notably, the synergistic effect of metabolic factors with inflammation and oxidative stress, which often results in a more rapid and significant escalation of RDW [39–41]. Accordingly, our research identified the most adverse outcomes in the cohort with rapidly increasing RDW, as evidenced by poor performance across several clinical indices, such as albumin, lactate, total bilirubin, sodium, and potassium levels. This aligns with the highest mortality risk associated with the “rapidly increasing” RDW trajectory group within our study. Additionally, Sepsis-induced endothelial dysfunction and compromised microcirculatory perfusion can precipitate recurrent mechanical injury or aggregation of red blood cells within constricted or compromised vascular networks, thereby augmenting the heterogeneity of red blood cell volume distribution. When red blood cell membrane fluidity and deformability are restricted, blood viscosity increases, exacerbating or maintaining a vicious cycle of systemic hypoxia and organ dysfunction, leading to sustained RDW elevation [42–44]. These studies indicated that the pathological basis of RDW is not merely inflammation-derived but involves the entire process of red blood cell generation and destruction, impacted by both internal and external environments. Future interventions targeting oxidative stress, improving nutritional status, and early correction of microcirculatory perfusion damage could potentially delay abnormal RDW elevation, thereby improving clinical outcomes in SA-AKI patients.

Our study has several strengths, Firstly, it is the inaugural investigation to incorporate a longitudinal data analysis framework (GBTM) to characterize dynamic changes in RDW among patients with SA-AKI. By shifting the focus from a single time point to trajectory-based analysis, our findings reveal nuanced risk stratification beyond static measurements. Secondly, the large sample size derived from a well-established critical care database (MIMIC-IV) both enriches statistical power and enhances the diversity of clinical characteristics in the study cohort, reinforcing the internal and external validity of our conclusions. Thirdly, a comprehensive multivariable adjustment and multiple imputation were carried out to reduce confounding and address potential biases due to missing data, thereby increasing the robustness and reliability of the observed associations. Fourthly, we undertook targeted sensitivity analyses to address key sources of bias: (i) relaxed inclusion criteria (≥3 RDW, no ICU LOS restriction) reproduced the 4-class structure and yielded consistent associations; (ii) adjustment for any ICU transfusion and any major hemorrhage, along with an analysis excluding transfused patients, minimally changed estimates; and (iii) models excluding severity scores produced similar results.

Despite several strengths, our study acknowledges certain limitations. Firstly, the research is based on the MIMIC-Ⅳ database, which is predominantly from a single-center ICU. While the sample is substantial and diverse, the database’s geographical, racial, and healthcare practice constraints may limit the generalizability of our findings. Secondly, our study is a retrospective analysis of the MIMIC-Ⅳ database. Although we controlled for confounding factors through multivariable adjustments and sensitivity analyses, the potential impact of unmeasured or unknown confounders cannot be entirely dismissed. In particular, hematologic interventions may influence RDW; however, adjusting for any ICU transfusion and any major hemorrhage and excluding transfused patients yielded similar estimates, suggesting limited confounding from these factors. Thirdly, selection and survivor biases may have arisen from requiring ≥5 RDW measurements and ICU LOS ≥ 96 h in the main cohort; to characterize and mitigate this, we compared included versus excluded/early-death patients (several variables had standardized differences >0.10), replicated analyses in a relaxed cohort (≥3 RDW) with consistent findings. Fourthly, although severity scores were computed within the first 24 hours (prior to trajectory assignment) to reduce post-exposure adjustment, residual time-dependent confounding remains possible; importantly, models excluding severity scores produced similar associations. Fifthly, measurement frequency and scheduling may introduce information bias; we mitigated this by retaining the first RDW per calendar day and by testing robustness in sensitivity cohorts. Finally, while RDW trajectories are associated with mortality, the retrospective design precludes causal inference; we therefore avoided causal language and interpret RDW as a dynamic prognostic indicator rather than a causal determinant. Future work should include multicenter prospective cohorts to bolster external validity; incorporate inflammatory and oxidative stress biomarkers to enhance mechanistic understanding; and evaluate whether trajectory-informed care pathways or targeted interventions can improve outcomes in high-risk (e.g., rapidly increasing) groups.

## Conclusion

In this study, we employed GBTM to identify four distinct trajectories of RDW dynamics and confirmed that an increasing RDW trajectory is independently associated all-cause mortality in patients with SA-AKI. Results were robust across subgroup analyses, multiple-imputation datasets, relaxed inclusion criteria, and models adjusting for or excluding transfusion and hemorrhage. The dynamic monitoring of RDW may provide a more precise basis for risk stratification in clinical settings and serve as a pragmatic prognostic indicator to inform personalized intervention strategies for critically ill patients, while acknowledging that causal effects cannot be inferred from this retrospective analysis.

## Supporting information

S1 File Tables S1–S10 and Fig S1: additional methods, missingness, trajectory model diagnostics, baseline comparisons, imputation diagnostics, sensitivity analyses, and RDW trajectories under relaxed inclusion criteria. (DOCX)

## Abbreviations

RDW: , Red cell distribution width;
SA-AKI: , Sepsis-associated acute kidney injury;
GBTM: , Group-Based Trajectory Modeling;
MAP: , Mean arterial pressure;
SpO2: , Saturation of peripheral oxygen;
SOFA: , Sequential organ failure assessment;
APS Ⅲ: , Acute physiology score Ⅲ;
OASIS: , Oxford acute severity of illness score;
CCI: , Charlson comorbidity index;
COPD: , Chronic obstructive pulmonary disease;
WBC: , White blood cell count;
PLT: , Platelet count;
tCa: , Total Calcium;
Tbil: , Total bilirubin;
CRRT: Continuous renal replacement therapy.

## References

[1] Pais T, Jorge S, Lopes JA. Acute kidney injury in sepsis. Int J Mol Sci. 2024;25. 10.3390/ijms25115924 PMC11172431 38892111

[2] Jensen SK, Heide-Jørgensen U, Gammelager H, Birn H, Christiansen CF. Acute kidney injury duration and 20-year risks of CKD and cardiovascular disease. Kidney Int Rep. 2024;9(4):817–29. doi: 10.1016/j.ekir.2024.01.034 38765592 PMC11101785

[3] Song MJ, Jang Y, Legrand M, Park S, Ko R, Suh GY, et al. Epidemiology of sepsis-associated acute kidney injury in critically ill patients: a multicenter, prospective, observational cohort study in South Korea. Crit Care. 2024;28(1):383. doi: 10.1186/s13054-024-05167-9 39581988 PMC11587587

[4] Zarbock A, Nadim MK, Pickkers P, Gomez H, Bell S, Joannidis M, et al. Sepsis-associated acute kidney injury: consensus report of the 28Th acute disease quality initiative workgroup. Nat Rev Nephrol. 2023;19:401–17. 36823168 10.1038/s41581-023-00683-3

[5] Vashistha T, Streja E, Molnar MZ, Rhee CM, Moradi H, Soohoo M, et al. Red cell distribution width and mortality in hemodialysis patients. Am J Kidney Dis. 68, 110–21 (2016). 26786297 10.1053/j.ajkd.2015.11.020 PMC4921311

[6] Huang B, Yan J, Li C, Jin F, Ma R, Cao G, et al. Red blood cell distribution width is a risk factor for multiple organ dysfunction syndrome in elderly patients with infection: a case control study. Aging Clin Exp Res. 2023;35(7):1577–80. doi: 10.1007/s40520-023-02431-w 37233902

[7] Urben T, Amacher SA, Becker C, Gross S, Arpagaus A, Tisljar K, et al. Red blood cell distribution width for the prediction of outcomes after cardiac arrest. Sci Rep. 2023;13(1):15081. doi: 10.1038/s41598-023-41984-8 37700019 PMC10497505

[8] Mercader-Salvans J, García-González M, Quevedo-Abeledo JC, Quevedo-Rodríguez A, Gómez-Bernal F, Hernández-Díaz M, et al. Red blood cell distribution width as a surrogate biomarker of damage and disease activity in patients with systemic lupus erythematosus. Clin Exp Rheumatol. 2024;42(9):1773–80. doi: 10.55563/clinexprheumatol/f0jnnm 38757296

[9] Feng J, Huang Y, Huang L, Zhao X, Li X, Xin A, et al. Association between RDW-SD and prognosis across glycemic status in patients with dilated cardiomyopathy. BMJ Open Diabetes Res Care. 2024;12(6):e004478. doi: 10.1136/bmjdrc-2024-004478 39542527 PMC11575278

[10] Zhang L, Yu C-H, Guo K-P, Huang C-Z, Mo L-Y. Prognostic role of red blood cell distribution width in patients with sepsis: a systematic review and meta-analysis. BMC Immunol. 2020;21(1):40. doi: 10.1186/s12865-020-00369-6 32631218 PMC7339553

[11] Kim CH, Park JT, Kim EJ, Han JH, Han JS, Choi JY, et al. An increase in red blood cell distribution width from baseline predicts mortality in patients with severe sepsis or septic shock. Crit Care. 2013;17(6):R282. doi: 10.1186/cc13145 24321201 PMC4056357

[12] Lai H, Wu G, Zhong Y, Chen G, Zhang W, Shi S, et al. Red blood cell distribution width improves the prediction of 28-day mortality for patients with sepsis-induced acute kidney injury: A retrospective analysis from MIMIC-IV database using propensity score matching. J Intensive Med. 2023;3(3):275–82. doi: 10.1016/j.jointm.2023.02.005 37533812 PMC10391576

[13] Jones BL, Nagin DS. Advances in group-based trajectory modeling and an sas procedure for estimating them. Sociol Methods Res. 2007;35:542–71.

[14] Bahorik AL, Hoang TD, Jacobs DR, Levine DA, Yaffe K. Association of changes in C-Reactive protein level trajectories through early adulthood with cognitive function at midlife: the CARDIA Study. Neurology. 2024;103(2):e209526. doi: 10.1212/WNL.0000000000209526 38959452 PMC11226328

[15] Johnson A, Bulgarelli L, Pollard T, Gow B, Moody B, Horng S, et al. MIMIC-IV (version 3.0). PhysioNet. 2024. Available from: https://physionet.org/content/mimiciv/3.0/

[16] Hu H, An S, Sha T, Wu F, Jin Y, Li L, et al. Association between dexmedetomidine administration and outcomes in critically ill patients with sepsis-associated acute kidney injury. J Clin Anesth. 2022;83:110960. doi: 10.1016/j.jclinane.2022.110960 36272399

[17] Zarbock A, et al. Sepsis-associated acute kidney injury: consensus report of the 28th acute disease quality initiative workgroup. Nat Rev Nephrol. 2023;19:401–17. 36823168 10.1038/s41581-023-00683-3

[18] Singer M, et al. The third international consensus definitions for sepsis and septic shock (Sepsis-3). JAMA. 2016;315:801. 26903338 10.1001/jama.2016.0287 PMC4968574

[19] Khwaja A. KDIGO clinical practice guidelines for acute kidney injury. Nephron Clin Pract. 2012;120(4):c179-84. doi: 10.1159/000339789 22890468

[20] De Rosa S, Samoni S, Ronco C. Creatinine-based definitions: from baseline creatinine to serum creatinine adjustment in intensive care. Crit Care. 2016;20:69. doi: 10.1186/s13054-016-1218-4 26983854 PMC4794949

[21] Xu X, Huang R, Lin Y, Guo Y, Xiong Z, Zhong X, et al. High triglyceride-glucose index in young adulthood is associated with incident cardiovascular disease and mortality in later life: insight from the CARDIA study. Cardiovasc Diabetol. 2022;21(1):155. doi: 10.1186/s12933-022-01593-7 35962377 PMC9375240

[22] van de Schoot R, Sijbrandij M, Winter SD, Depaoli S, Vermunt JK. The grolts-checklist: guidelines for reporting on latent trajectory studies. Struct Equ Model. 2017;24:451–67.

[23] Rubin DB. Multiple imputation for nonresponse in surveys. New York, NY: John Wiley & Sons Inc; 1987.

[24] van Kimmenade RRJ, Mohammed AA, Uthamalingam S, van der Meer P, Felker GM, Januzzi JL Jr. Red blood cell distribution width and 1-year mortality in acute heart failure. Eur J Heart Fail. 2010;12(2):129–36. doi: 10.1093/eurjhf/hfp179 20026456

[25] Zhang B, Han X, Long W, Xi S, Yu B, Yuan X. Association between red blood cell distribution width in late pregnancy and the incidence of adverse perinatal outcomes: a retrospective cohort study. Arch Med Res. 2024;55(7):103057. doi: 10.1016/j.arcmed.2024.103057 39067407

[26] Huo L, Zhao W, Ji X, Chen K, Liu T. The combination effect of the red blood cell distribution width and prognostic nutrition index on the prognosis in patients undergoing PCI. Nutrients. 2024;16(18):3176. doi: 10.3390/nu16183176 39339776 PMC11434894

[27] Wu Y-C, Chen H-H, Chao W-C. Association between red blood cell distribution width and 30-day mortality in critically ill septic patients: a propensity score-matched study. J Intensive Care. 2024;12(1):34. doi: 10.1186/s40560-024-00747-x 39294760 PMC11409593

[28] Ji X, Ke W. Red blood cell distribution width and all-cause mortality in congestive heart failure patients: a retrospective cohort study based on the Mimic-III database. Front Cardiovasc Med. 2023;10:1126718. doi: 10.3389/fcvm.2023.1126718 37206106 PMC10189655

[29] Hu Z-D, Wei T-T, Tang Q-Q, Fu H-T, Yang M, Ma N, et al. Prognostic value of red blood cell distribution width in acute pancreatitis patients admitted to intensive care units: an analysis of a publicly accessible clinical database MIMIC II. Clin Chem Lab Med. 2016;54(7):e195-7. doi: 10.1515/cclm-2015-1021 26812874

[30] Wu H, Liao B, Cao T, Ji T, Huang J, Ma K. Diagnostic value of RDW for the prediction of mortality in adult sepsis patients: A systematic review and meta-analysis. Front Immunol. 2022;13:997853. doi: 10.3389/fimmu.2022.997853 36325342 PMC9618606

[31] Vanderelst J, Rousseau A, Selvais N, Biston P, Zouaoui Boudjeltia K, Piagnerelli M. Evolution of red blood cell membrane complement regulatory proteins and rheology in septic patients: an exploratory study. Front Med (Lausanne). 2022;9:880657. doi: 10.3389/fmed.2022.880657 35966861 PMC9366164

[32] Pernow J, Mahdi A, Yang J, Zhou Z. Red blood cell dysfunction: a new player in cardiovascular disease. Cardiovasc Res. 2019;115(11):1596–605. doi: 10.1093/cvr/cvz156 31198931

[33] Becatti M, et al. Erythrocyte membrane fluidity alterations in sudden sensorineural hearing loss patients: The role of oxidative stress. Thromb Haemost. 2017;117:2334–45. 29212121 10.1160/TH17-05-0356

[34] Kozlova E, et al. Mechanochemical synergism of reactive oxygen species influences on Rbc membrane. Int J Mol Sci. 2023;24:5952. 36983026 10.3390/ijms24065952 PMC10057059

[35] Esperti S, Nader E, Stier A, Boisson C, Carin R, Marano M, et al. Increased retention of functional mitochondria in mature sickle red blood cells is associated with increased sickling tendency, hemolysis and oxidative stress. Haematologica. 2023;108(11):3086–94. doi: 10.3324/haematol.2023.282684 37259576 PMC10620576

[36] Zhang D-L, Ghosh MC, Ollivierre H, Li Y, Rouault TA. Ferroportin deficiency in erythroid cells causes serum iron deficiency and promotes hemolysis due to oxidative stress. Blood. 2018;132(19):2078–87. doi: 10.1182/blood-2018-04-842997 30213870 PMC6236465

[37] Matsuoka T, Abe M, Kobayashi H. Iron metabolism and inflammatory mediators in patients with renal dysfunction. Int J Mol Sci. 2024;25. 10.3390/ijms25073745 PMC11012052 38612557

[38] Maras JS, Maiwall R, Harsha HC, Das S, Hussain MS, Kumar C, et al. Dysregulated iron homeostasis is strongly associated with multiorgan failure and early mortality in acute-on-chronic liver failure. Hepatology. 2015;61(4):1306–20. doi: 10.1002/hep.27636 25475192

[39] Biswas SK. Does the interdependence between oxidative stress and inflammation explain the antioxidant paradox? Oxid Med Cell Longev. 2016;2016:5698931. doi: 10.1155/2016/5698931 26881031 PMC4736408

[40] Pearce KL, Hill A, Tremellen KP. Obesity related metabolic endotoxemia is associated with oxidative stress and impaired sperm DNA integrity. Basic Clin Androl. 2019;29:6. doi: 10.1186/s12610-019-0087-5 31114691 PMC6513521

[41] Tobore TO. Towards a comprehensive theory of obesity and a healthy diet: the causal role of oxidative stress in food addiction and obesity. Behav Brain Res. 2020;384:112560. doi: 10.1016/j.bbr.2020.112560 32081711

[42] De Backer D, Orbegozo Cortes D, Donadello K, Vincent J-L. Pathophysiology of microcirculatory dysfunction and the pathogenesis of septic shock. Virulence. 2014;5(1):73–9. doi: 10.4161/viru.26482 24067428 PMC3916386

[43] McMahon TJ. Red blood cell deformability, vasoactive mediators, and adhesion. Front Physiol. 2019;10:1417. doi: 10.3389/fphys.2019.01417 31803068 PMC6873820

[44] Baudry N, Starck J, Aussel C, Lund K, Aletti M, Duranteau J, et al. Effect of preconditioned mesenchymal stromal cells on early microvascular disturbance in a mouse sepsis model. Stem Cells Dev. 2019;28(24):1595–606. doi: 10.1089/scd.2019.0134 31663453

