# Supplementary material for: Longitudinal modeling of red blood cell distribution width dynamics and mortality risk in critically Ill patients with sepsis-associated acute kidney injury
Source: PLoS One. 2025 Oct 8;20(10):e0333605. doi: 10.1371/journal.pone.0333605 (PMC12507301; doi:10.1371/journal.pone.0333605)
Supplement: S1 File — (DOCX) [file pone.0333605.s001.docx]

**Supplementary Material**

**Supplementary Table S1. Percentage of missing data in variables of interest.**

| **Variable** | **Miss. frequency (n)** | **Miss. percentage%** |
| --- | --- | --- |
| Albumin (g/dL) | 1590 | 23.7526 |
| BMI (kg/m2) | 1313 | 19.6146 |
| Total bilirubin (mg/dL) | 1089 | 16.2683 |
| Lactate (mmol/L) | 506 | 7.559 |
| PH | 324 | 4.8402 |
| MAP (mmHg) | 43 | 0.6424 |
| Hematocrit (%) | 36 | 0.5378 |
| Platelet count (k/μL) | 32 | 0.478 |
| RDW (%) | 31 | 0.4631 |
| Hemoglobin (g/dL) | 27 | 0.4033 |
| WBC (k/μL) | 27 | 0.4033 |
| Potassium (mmol/L) | 8 | 0.1195 |
| Bicarbonate (mmol/L) | 6 | 0.0896 |
| Total calcium (mg/dL) | 3 | 0.0448 |
| Chloride (mmol/L) | 1 | 0.0149 |
| Creatinine (mg/dL) | 1 | 0.0149 |
| Sodium (mmol/L) | 1 | 0.0149 |

**Supplementary Table S2. Comparison of Evaluation Indicators for Group-based Trajectory Modeling Across Groups Ranging from Two to Five.**

| **Polynomial**  **degree** | **Latent**  **classes** | **Log**  **likelihood** | **AIC** | **BIC** | **SABIC** | **entropy** |
| --- | --- | --- | --- | --- | --- | --- |
| Linear |  |  |  |  |  |  |
|  | 2 | -113938.26 | 227896.51 | 227964.60 | 227932.83 | 0.62 |
|  | 3 | -113595.84 | 227219.68 | 227315.00 | 227270.51 | 0.57 |
|  | 4 | -113445.83 | 226927.66 | 227050.22 | 226993.02 | 0.58 |
|  | 5 | -113395.04 | 226834.08 | 226983.88 | 226913.97 | 0.60 |
| Quadratic |  |  |  |  |  |  |
|  | 2 | -120143.19 | 240306.38 | 240374.47 | 240342.69 | 0.65 |
|  | 3 | -119557.23 | 239142.47 | 239237.79 | 239193.31 | 0.64 |
|  | 4 | -119340.42 | 238716.84 | 238839.40 | 238782.21 | 0.61 |
|  | 5 | -119231.50 | 238507.00 | 238656.80 | 238586.89 | 0.60 |
| Cubic |  |  |  |  |  |  |
|  | 2 | -127408.17 | 254836.33 | 254904.42 | 254872.64 | 0.77 |
|  | 3 | -126492.12 | 253012.23 | 253107.56 | 253063.07 | 0.67 |
|  | 4 | -126210.39 | 252456.78 | 252579.34 | 252522.14 | 0.63 |
|  | 5 | -126049.71 | 252143.42 | 252293.22 | 252223.31 | 0.63 |

AIC, Akaike information criterion; BIC, Bayesian information criteria; SABIC, Sample-adjusted information criteria

**Supplementary Table S3. Comparison of Posterior classifications for Group-based Trajectory Modeling Across Groups Ranging from Two to Five.**

| **Polynomial degree** | **Latent classes** | **Participants per class (%)** | **Posterior probabilities** |
| --- | --- | --- | --- |
| Linear |  |  |  |
|  | 2 | 74.71/25.29 | 0.9/0.9 |
|  | 3 | 54.17/9.04/36.79 | 0.82/0.87/0.78 |
|  | **4** | **27.8/38.45/27.64/6.11** | **0.79/0.71/0.79/0.86** |
|  | 5 | 8.66/29.94/31.83/5.39/24.17 | 0.75/0.69/0.70/0.87/0.79 |
| Quadratic |  |  |  |
|  | 2 | 78.62/21.38 | 0.91/0.91 |
|  | 3 | 63.85/4.51/31.64 | 0.85/0.87/0.84 |
|  | 4 | 35.82/36.48/3.33/24.37 | 0.8/0.72/0.85/0.85 |
|  | 5 | 28.61/34.03/26.31/1.34/9.71 | 0.8/0.68/0.74/0.84/0.81 |
| Cubic |  |  |  |
|  | 2 | 87.6/12.4 | 0.95/0.9 |
|  | 3 | 68.05/28.25/3.7 | 0.85/0.86/0.89 |
|  | 4 | 43.35/33.09/20.99/2.57 | 0.82/0.73/0.87/0.9 |
|  | 5 | 31.01/24.08/33.24/9.99/1.67 | 0.82/0.75/0.7/0.83/0.87 |

**Supplementary Table S4. Trajectory model selection metrics. Bayesian Information Criterion, Akaike Information Criterion, entropy, and minimum class proportions for 2–5 class solutions (≥3 RDW measurements).**

| **Latent**  **classes** | **AIC** | **BIC** | **SABIC** | **entropy** | **Participants per class (%)** | **Posterior probabilities** |
| --- | --- | --- | --- | --- | --- | --- |
|  | | | | | | |
| 2 | 357992.59 | 358068.88 | 358037.10 | 0.60 | 75.73/24.27 | 0.89/0.9 |
| 3 | 356525.10 | 356631.90 | 356587.41 | 0.57 | 56.21/8.07/35.71 | 0.82/0.86/0.78 |
| **4** | **355925.63** | **356062.94** | **356005.74** | **0.57** | **33.13/38.24/24.21/4.42** | **0.78/0.7/0.79/0.85** |
| 5 | 355756.32 | 355924.15 | 355854.24 | 0.56 | 13.87/30.5/31.67/3.65/20.32 | 0.71/0.69/0.65/0.85/0.79 |

AIC, Akaike information criterion; BIC, Bayesian information criteria; SABIC, Sample-adjusted information criteria

**Supplementary Table S5. Baseline comparison of included (≥5 RDW) vs excluded/early-death patients (<5 RDW or ICU LOS ≤96 h), with standardized differences.**

| Variables | Group 1  (n = 6694) | Group 2  (n = 9963) | Standardized Difference |
| --- | --- | --- | --- |
| Age (years) | 65.5 ± 16.3 | 68.8 ± 15.6 | 0.207 |
| Male (%) | 3865 (57.7) | 5882 (59) | 0.026 |
| Race_white (%) | 4159 (62.1) | 6858 (68.8) | 0.141 |
| BMI (kg/m^2^) | 30.1 ± 8.5 | 30.0 ± 7.4 | 0.013 |
| MAP (mmHg) | 85.63 ± 18.8 | 83.73 ± 17.63 | 0.104 |
| SOFA | 6.8 ± 3.7 | 5.8 ± 3.4 | 0.281 |
| APS Ⅲ | 55.0 ± 22.5 | 49.1 ± 22.5 | 0.262 |
| OASIS | 36.8 ± 8.2 | 34.2 ± 8.3 | 0.315 |
| CCI | 5.2 ± 2.9 | 5.3 ± 2.9 | 0.034 |
| Hypertension | 2790 (41.7) | 4408 (44.2) | 0.052 |
| Diabetes mellitus | 2041 (30.5) | 3152 (31.6) | 0.025 |
| Heart failure | 2116 (31.6) | 2719 (27.3) | 0.095 |
| Malignant tumors | 919 (13.7) | 1750 (17.6) | 0.106 |
| COPD | 656 (9.8) | 757 (7.6) | 0.078 |
| Cirrhosis | 641 (9.6) | 917 (9.2) | 0.013 |
| WBC (k/μL) | 13.64 ± 8.31 | 13.12 ± 7.81 | 0.064 |
| Hemoglobin (g/dL) | 10.8 ± 2.3 | 10.5 ± 2.2 | 0.133 |
| Hematocrit (%) | 32.9 ± 7.0 | 31.8 ± 6.5 | 0.163 |
| RDW (%) | 15.2 ± 2.3 | 15.0 ± 2.3 | 0.087 |
| PLT (k/μL) | 203.3 ± 111.6 | 191.5 ± 105.1 | 0.109 |
| Albumin (g/dL) | 2.9 ± 0.6 | 3.0 ± 0.6 | 0.167 |
| Sodium (mmol/L) | 138.6 ± 5.8 | 138.5 ± 5.4 | 0.018 |
| Potassium (mmol/L) | 4.2 ± 0.8 | 4.3 ± 0.8 | 0.125 |
| tCa (mg/dL) | 8.2 ± 0.9 | 8.2 ± 0.8 | 0 |
| Chloride (mmol/L) | 104.3 ± 7.1 | 105.0 ± 6.8 | 0.101 |
| Lactate (mmol/L) | 2.46 ± 2.05 | 2.56 ± 2.21 | 0.047 |
| Tbil (mg/dL) | 1.72 ± 3.77 | 2.07 ± 4.33 | 0.086 |
| Creatinine (mg/dL) | 1.49 ± 1.44 | 1.52 ± 1.62 | 0.02 |
| Bicarbonate (mmol/L) | 22.3 ± 5.1 | 22.3 ± 4.7 | 0 |

Note:

Group 1: Included patients (≥5 RDW).

Group 2: Excluded/early-death patients (<5 RDW or ICU LOS ≤96 h).

**Supplementary Table S6. Comparing the distribution of data before and after multiple imputation.**

| **Variables** | **Before imputation** | **Imputation 1** | **Imputation 2** | **Imputation 3** | **Imputation 4** | **Imputation 5** | ***P*-value** |
| --- | --- | --- | --- | --- | --- | --- | --- |
| Albumin (g/dL) | 2.9 ± 0.6 | 2.9 ± 0.6 | 2.9 ± 0.6 | 2.9 ± 0.6 | 2.9 ± 0.6 | 2.9 ± 0.6 | 0.672 |
| BMI (kg/m2) | 30.1 ± 8.5 | 30.1 ± 8.4 | 30.2 ± 8.5 | 30.1 ± 8.5 | 30.2 ± 8.5 | 30.1 ± 8.4 | 0.874 |
| Total bilirubin (mg/dL) | 0.7 (0.4, 1.4) | 0.7 (0.4, 1.3) | 0.7 (0.4, 1.3) | 0.7 (0.4, 1.3) | 0.7 (0.4, 1.3) | 0.7 (0.4, 1.3) | 0.508 |
| Lactate (mmol/L) | 1.8 (1.2, 2.9) | 1.8 (1.2, 2.9) | 1.8 (1.2, 2.9) | 1.8 (1.2, 2.9) | 1.8 (1.2, 2.9) | 1.8 (1.2, 2.9) | 0.937 |
| PH | 7.3 ± 0.1 | 7.3 ± 0.1 | 7.3 ± 0.1 | 7.3 ± 0.1 | 7.3 ± 0.1 | 7.3 ± 0.1 | 0.978 |
| MAP (mmHg) | 83.7(72.3, 97.0) | 83.7(72.3, 97.0) | 83.3(72.3, 97.0) | 83.5(72.3, 97.0) | 83.7(72.3, 97.0) | 83.7(72.3, 97.0) | 1 |
| Hematocrit (%) | 32.9 ± 7.0 | 33.0 ± 7.0 | 32.9 ± 7.0 | 33.0 ± 7.0 | 32.9 ± 7.0 | 32.9 ± 7.0 | 1 |
| Platelet count (k/μL) | 203.3 ± 111.6 | 203.3 ± 111.8 | 203.2 ± 111.6 | 203.3 ± 111.7 | 203.2 ± 111.6 | 203.2 ± 111.7 | 1 |
| RDW (%) | 15.2 ± 2.3 | 15.2 ± 2.3 | 15.2 ± 2.3 | 15.2 ± 2.3 | 15.2 ± 2.3 | 15.2 ± 2.3 | 1 |
| Hemoglobin (g/dL) | 10.8 ± 2.3 | 10.8 ± 2.3 | 10.8 ± 2.3 | 10.8 ± 2.3 | 10.8 ± 2.3 | 10.8 ± 2.3 | 1 |
| WBC (k/μL) | 12.1 (8.6, 16.9) | 12.1 (8.5, 16.9) | 12.2 (8.6, 16.9) | 12.1 (8.5, 16.9) | 12.1 (8.6, 16.9) | 12.2 (8.6, 16.9) | 1 |
| Potassium (mmol/L) | 4.2 ± 0.8 | 4.2 ± 0.8 | 4.2 ± 0.8 | 4.2 ± 0.8 | 4.2 ± 0.8 | 4.2 ± 0.8 | 1 |
| Bicarbonate (mmol/L) | 22.3 ± 5.1 | 22.3 ± 5.1 | 22.3 ± 5.1 | 22.3 ± 5.1 | 22.3 ± 5.1 | 22.3 ± 5.1 | 1 |
| Total calcium (mg/dL) | 8.2 ± 0.9 | 8.2 ± 0.9 | 8.2 ± 0.9 | 8.2 ± 0.9 | 8.2 ± 0.9 | 8.2 ± 0.9 | 1 |
| Chloride (mmol/L) | 104.3 ± 7.1 | 104.3 ± 7.1 | 104.3 ± 7.1 | 104.3 ± 7.1 | 104.3 ± 7.1 | 104.3 ± 7.1 | 1 |
| Creatinine (mg/dL) | 1.0 (0.8, 1.6) | 1.0 (0.8, 1.6) | 1.0 (0.8, 1.6) | 1.0 (0.8, 1.6) | 1.0 (0.8, 1.6) | 1.0 (0.8, 1.6) | 1 |
| Sodium (mmol/L) | 138.6 ± 5.8 | 138.6 ± 5.8 | 138.6 ± 5.8 | 138.6 ± 5.8 | 138.6 ± 5.8 | 138.6 ± 5.8 | 1 |

**Supplementary Table S7. Multivariable Cox regression analyses for 28-day all-cause mortality according to RDW trajectories，before and after using multiple imputation for missing data.**

|  | **Unadjusted Model** | | **Adjusted Model*** | |
| --- | --- | --- | --- | --- |
| **Categories** | **HR (95%CI)** | ***P*-value** | **HR (95%CI)** | ***P*-value** |
| **Before imputation** |  |  |  |  |
| Stable Low group | 1(Ref) |  | 1(Ref) |  |
| Gradual Increase group | 1.55 (1.32-1.81) | <0.001 | 1.45 (1.14-1.83) | 0.002 |
| Continuous Increase group | 2.64 (2.27-3.07) | <0.001 | 2.18 (1.71-2.78) | <0.001 |
| Rapid Increase group | 5.47 (4.55-6.57) | <0.001 | 4.27 (3.18-5.75) | <0.001 |
| **After imputation** |  |  |  |  |
| Stable Low group | 1(Ref) |  | 1(Ref) |  |
| Gradual Increase group | 1.55 (1.32-1.81) | <0.001 | 1.34(1.14, 1.58) | <0.001 |
| Continuous Increase group | 2.64 (2.27-3.07) | <0.001 | 1.93(1.62, 2.29) | <0.001 |
| Rapid Increase group | 5.47 (4.55-6.57) | <0.001 | 3.55(2.84, 4.44) | <0.001 |

* Results were adjusted for age, gender, race, BMI, MAP, SOFA, APS Ⅲ, OASIS, CCI, hypertension, diabetes mellitus, heart failure, malignant tumors, COPD, cirrhosis, WBC, hemoglobin, hematocrit, PLT, albumin, sodium, potassium, total calcium, chloride, PH, lactate, total bilirubin, creatinine, and bicarbonate.

**Supplementary Table S8. Cox regression results for ≥3 RDW sensitivity cohort (28-day and 90-day mortality).**

| **Categories** | **Model 1** | | **Model 2** | |
| --- | --- | --- | --- | --- |
|  | **HR (95%CI)** | ***P*-value** | **HR (95%CI)** | ***P*-value** |
| **28-day all-cause mortality** | | | | |
| Stable Low group | 1(Ref) |  | 1(Ref) |  |
| Gradual Increase group | 2.01 (1.78~2.26) | <0.001 | 1.42 (1.17~1.73) | <0.001 |
| Continuous Increase group | 3.77 (3.35~4.24) | <0.001 | 2.02 (1.65~2.48) | <0.001 |
| Rapid Increase group | 7.65 (6.6~8.87) | <0.001 | 3.89 (3.01~5.01) | <0.001 |
| **90-day all-cause mortality** | | | | |
| Stable Low group | 1(Ref) |  | 1(Ref) |  |
| Gradual Increase group | 2.01 (1.82~2.22) | <0.001 | 1.44 (1.21~1.71) | <0.001 |
| Continuous Increase group | 3.98 (3.6~4.39) | <0.001 | 2.05 (1.71~2.45) | <0.001 |
| Rapid Increase group | 7.28 (6.38~8.3) | <0.001 | 3.67 (2.93~4.61) | <0.001 |

Model 1: crude relative risk;

Model 2: adjusted for age, gender, race, BMI, MAP, SOFA, APS Ⅲ, OASIS, CCI, hypertension, diabetes mellitus, heart failure, malignant tumors, COPD, cirrhosis, WBC, hemoglobin, hematocrit, PLT, albumin, sodium, potassium, total calcium, chloride, PH, lactate, total bilirubin, creatinine, and bicarbonate.

**Supplementary Table S9. Cox proportional hazards analyses for adjusted for transfusion/hemorrhage and analysis excluding transfused patients.**

| **Categories** | **Model A (n=6694)** | | **Model B (n=6694)** | | **Model C (n=5199)** | |
| --- | --- | --- | --- | --- | --- | --- |
|  | **HR (95%CI)** | ***P*-value** | **HR (95%CI)** | ***P*-value** | **HR (95%CI)** | ***P*-value** |
| **28-day all-cause mortality** | | | | | | |
| Stable Low group | 1(Ref) |  | 1(Ref) |  | 1(Ref) |  |
| Gradual Increase group | 1.45 (1.14-1.83) | 0.002 | 1.46 (1.15~1.85) | 0.002 | 1.45 (1.13~1.88) | 0.004 |
| Continuous Increase group | 2.18 (1.71-2.78) | <0.001 | 2.2 (1.72~2.81) | <0.001 | 1.96 (1.5~2.58) | <0.001 |
| Rapid Increase group | 4.27 (3.18-5.75) | <0.001 | 4.29 (3.18~5.79) | <0.001 | 4.07 (2.83~5.86) | <0.001 |
| **90-day all-cause mortality** | | | | | | |
| Stable Low group | 1(Ref) |  | 1(Ref) |  | 1(Ref) |  |
| Gradual Increase group | 1.43 (1.16-1.75) | 0.001 | 1.43 (1.17~1.75) | 0.001 | 1.4 (1.13~1.75) | 0.002 |
| Continuous Increase group | 2.08 (1.69-2.56) | <0.001 | 2.07 (1.68~2.56) | <0.001 | 1.93 (1.53~2.44) | <0.001 |
| Rapid Increase group | 3.73 (2.87-4.85) | <0.001 | 3.71 (2.85~4.83) | <0.001 | 3.18 (2.29~4.42) | <0.001 |

Model A (main model without transfusion/hemorrhage covariates): adjusted for age, gender, race, BMI, MAP, SOFA, APS Ⅲ, OASIS, CCI, hypertension, diabetes mellitus, heart failure, malignant tumors, COPD, cirrhosis, WBC, hemoglobin, hematocrit, PLT, albumin, sodium, potassium, total calcium, chloride, PH, lactate, total bilirubin, creatinine, and bicarbonate.

Model B (with transfusion and hemorrhage covariates): further adjusted (from Model A) for transfusion and hemorrhage.

Model C (excluding all transfused patients): The adjusted variables are consistent with Model A.

**Supplementary Table S10. Cox proportional hazards analyses excluding severity scores (SOFA/APS III/OASIS).**

| **Categories** | **Model 1** | | **Model 2** | |
| --- | --- | --- | --- | --- |
|  | **HR (95%CI)** | ***P*-value** | **HR (95%CI)** | ***P*-value** |
| **28-day all-cause mortality** | | | | |
| Stable Low group | 1(Ref) |  | 1(Ref) |  |
| Gradual Increase group | 1.55 (1.32-1.81) | <0.001 | 1.47 (1.16~1.86) | 0.002 |
| Continuous Increase group | 2.64 (2.27-3.07) | <0.001 | 2.39 (1.88~3.05) | <0.001 |
| Rapid Increase group | 5.47 (4.55-6.57) | <0.001 | 4.75 (3.54~6.37) | <0.001 |
| **90-day all-cause mortality** | | | | |
| Stable Low group | 1(Ref) |  | 1(Ref) |  |
| Gradual Increase group | 1.59 (1.39~1.82) | <0.001 | 1.44 (1.17~1.76) | <0.001 |
| Continuous Increase group | 2.79 (2.45~3.19) | <0.001 | 2.28 (1.85~2.81) | <0.001 |
| Rapid Increase group | 5.16 (4.37~6.1) | <0.001 | 4.11 (3.17~5.33) | <0.001 |

Model 1: crude relative risk;

Model 2: adjusted for age, gender, race, BMI, MAP, hypertension, diabetes mellitus, heart failure, malignant tumors, COPD, cirrhosis, WBC, hemoglobin, hematocrit, PLT, albumin, sodium, potassium, total calcium, chloride, PH, lactate, total bilirubin, creatinine, and bicarbonate.


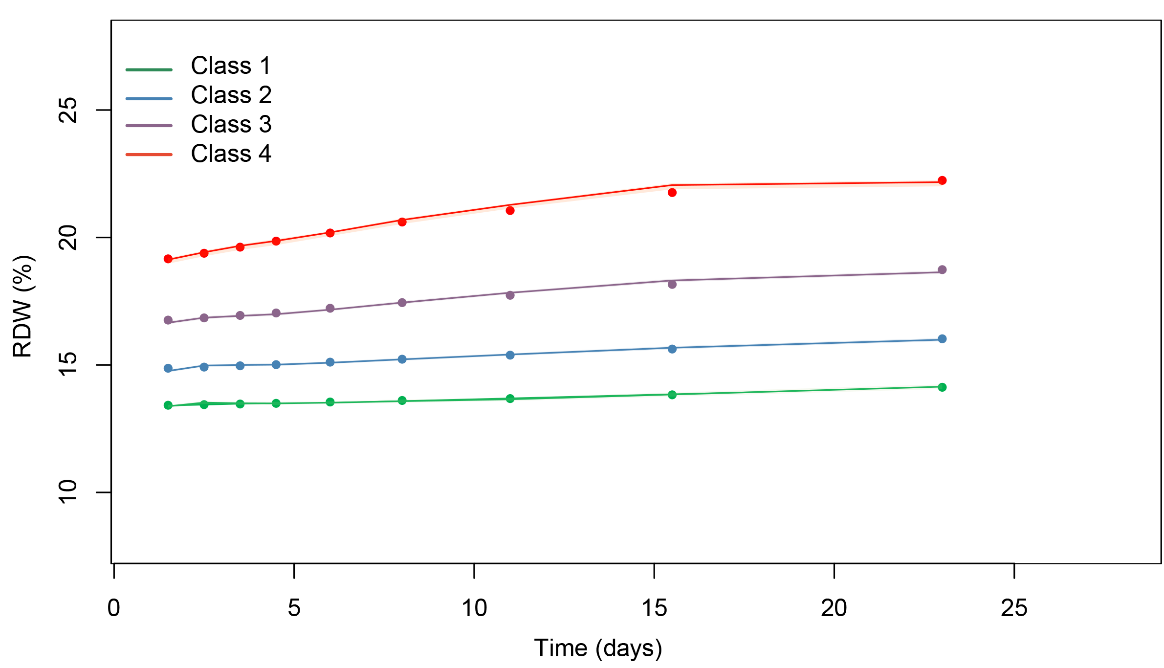


**Figure S1.** RDW trajectories under relaxed inclusion criteria.
